# Supplementary material for: The Arabidopsis AAC Proteins CIL and CIA2 Are Sub-functionalized Paralogs Involved in Chloroplast Development
Source: Front Plant Sci. 2021 Jun 7;12:681375. doi: 10.3389/fpls.2021.681375 (PMC8215611; doi:10.3389/fpls.2021.681375)
Supplement: Supplementary file 1 [file Data_Sheet_1.docx]

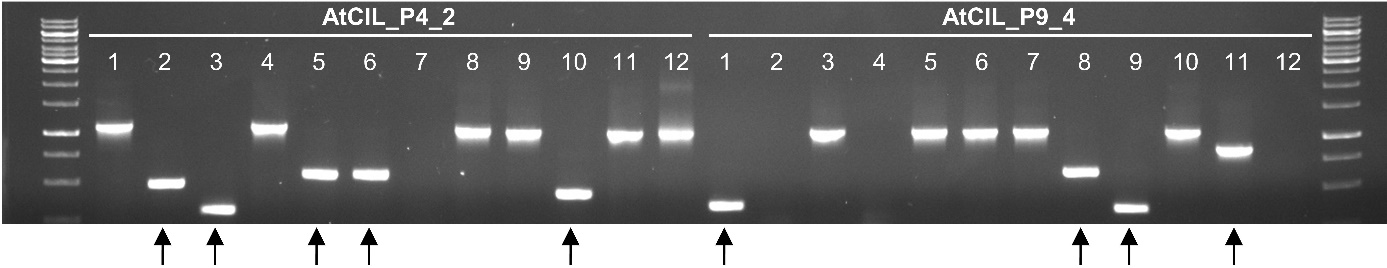


**Supplemental Figure 1. Mutation detection in T_1_ plants by colony-PCR.** First round PCR was performed using genomic DNA of T_1_ plants AtCIL_P4_2 and AtCIL _P9_4, respectively. PCR products were cloned into pJET1.2/blunt vector and fragment size of individual PCR product was revealed by colony-PCR. Twelve colonies were picked for each line. Arrows indicate PCR product carrying large deletions within the *cil* gene. The O’GeneRuler 1 kb DNA Ladder was loaded on both sides of the gel.


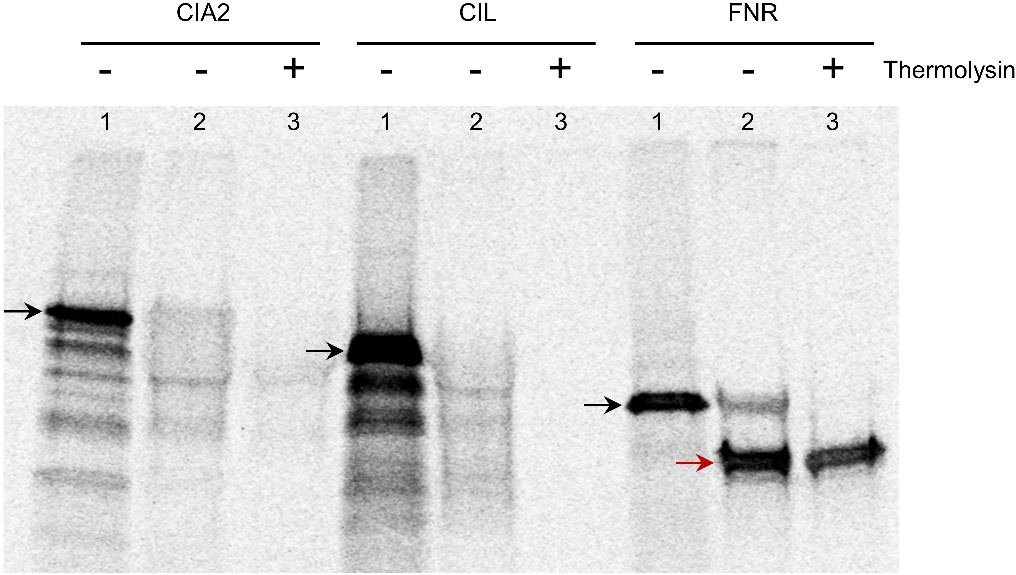


**Supplemental Figure 2. Chloroplast import assay.** Full-length proteins translated and radiolabeled in reticulocyte lysate (lane 1). *In vitro*-synthesized, radiolabeled proteins then were incubated with isolated pea chloroplasts. Subsequently, chloroplasts were incubated without (-, lane 2) or with (+, lane 3) the protease thermolysin. Precursor protein and mature protein are indicated by black and red arrows, respectively. A 10% of radiolabeled translation product used in the import reaction (lane 1). Chloroplast-localized FNR as input control. CIA2, CHLOROPLAST IMPORT APPARATUS 2; CIL, CHLOROPLAST IMPORT APPARATUS 2-LIKE; FNR, FERREDOXIN-NADP(+) REDUCTASE.


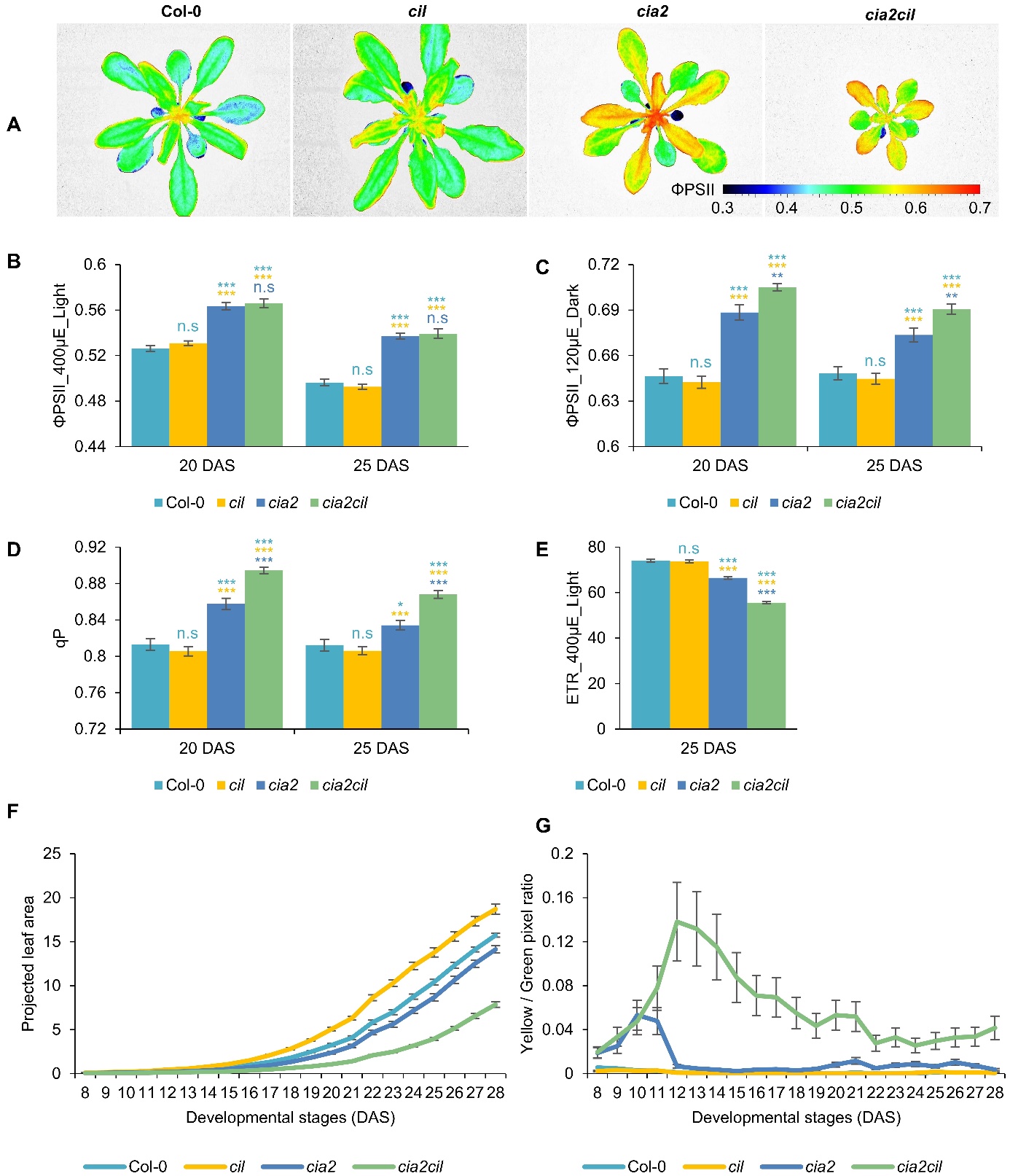


**Supplemental Figure 3. Measurement of photosynthetic performance of wild type Col-0 and mutants *cil*, *cia2* and *cia2cil*.**

(A) False color images of the operating light use efficiency of PSII (ΦPSII) at 400 µE actinic light.

(B-E) Measurement of photosynthetic parameters. Results are presented as mean ± SEM (N = 15). *Student’s t-test* (Tails =2; Type = 2) significant levels, n.s, not significant, * *p <* 0.05, * * *p <* 0.01, * * * *p <* 0.001. From top to bottom: statistical analysis of significance was performed as compared to Col-0, *cil* and *cia2*, respectively. ΦPSII_400µE_Light, Photosystem II operating efficiency of light-adapted plants at 400 µE; ΦPSII_120µE_Dark, Photosystem II operating efficiency of dark-adapted plants (i.e., 4 min following dark incubation) at 120 µE; qP, fraction of PSII reaction centers that are ‘open’ based on the puddle model; ETR_400µE_Light, electron transport rate of light-adapted plants at 400 µE. DAS, days after sowing.

(F-G) Plant growth dynamics. Left panel is projected leaf area (F) and right panel is yellow/green pixel ratio (G).


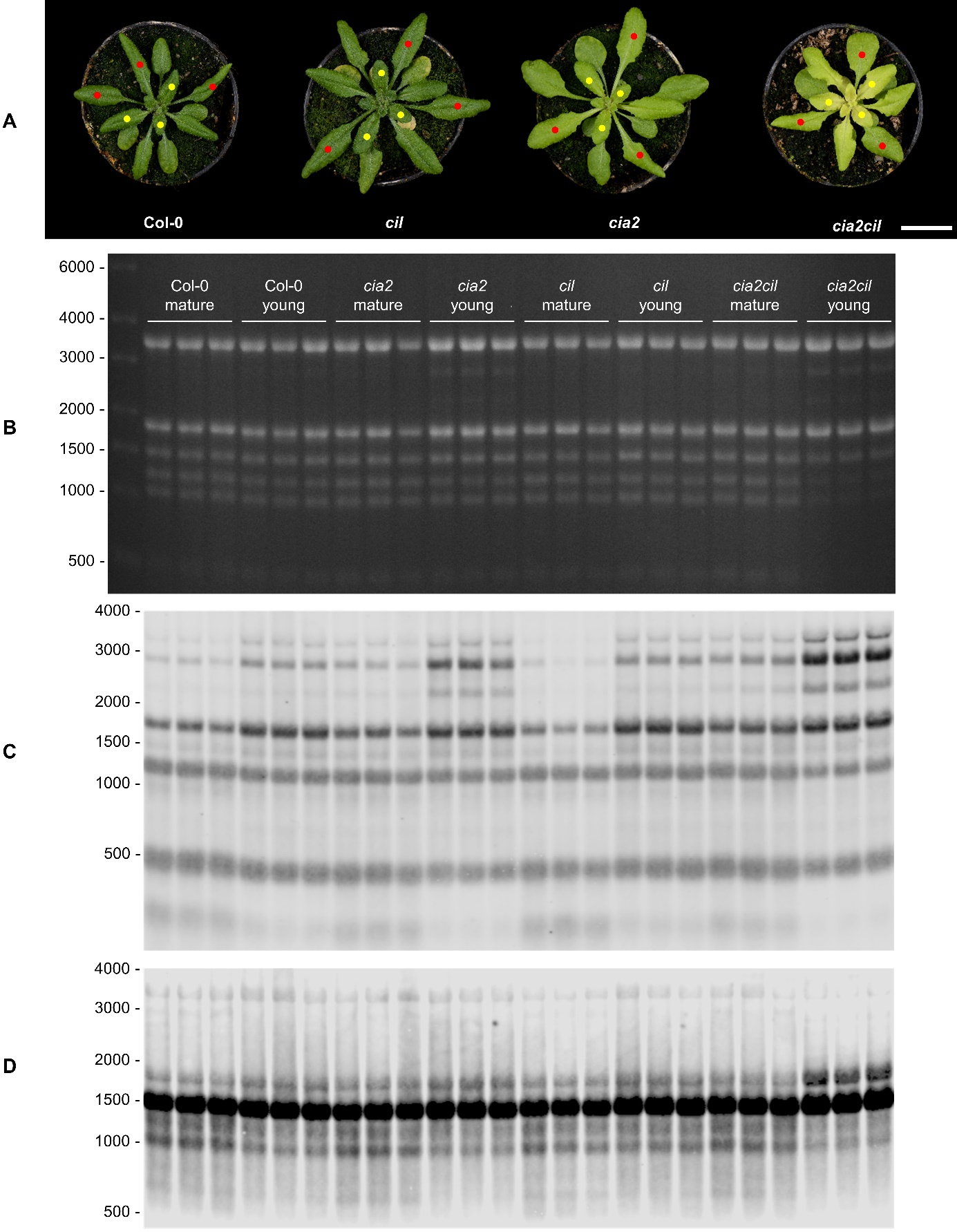


**Supplemental Figure 4. Analysis of chloroplast rRNA processing in Col-0, *cil*, *cia2* and *cia2cil* mutant plants.**

(A) Illustration of sample collection. The red dots represent the sampled ‘mature leaf’ material; the yellow dots indicate the ‘young leaf’. Scale bar, 2 cm.

(B) Separation of the cytosolic and chloroplastic rRNAs on agarose gel.

(C) Analysis of 23S rRNA processing by RNA gel-blot hybridization.

(D) Analysis of 16S rRNA processing by RNA gel-blot hybridization.


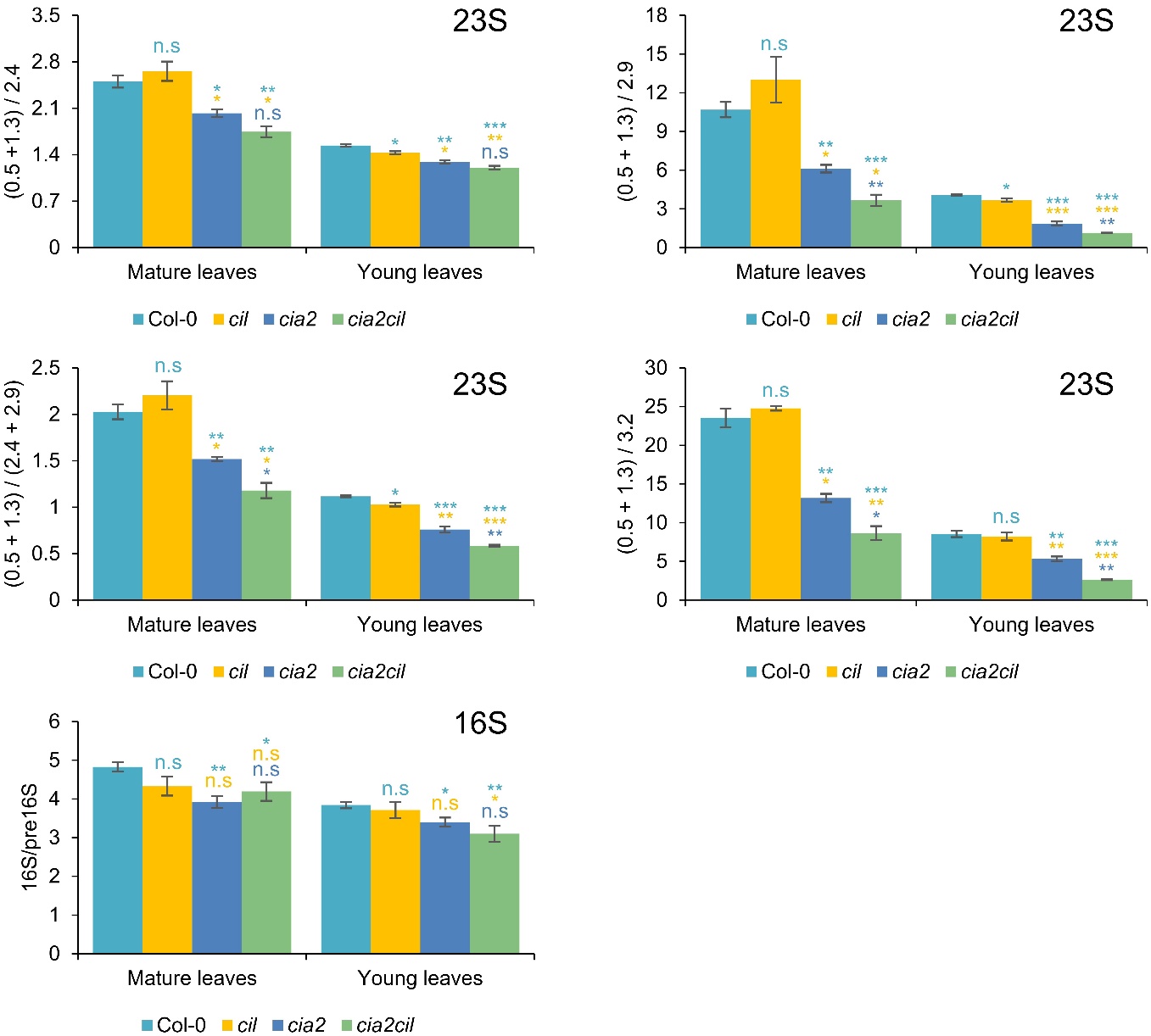


**Supplemental Figure 5. Quantification of ratio of mature 16S and 23S rRNA to pre-mature 16S and 23S rRNA species.** Results are presented as mean ± SEM (N=3). From top to bottom: statistical analysis of significance was performed as compared to Col-0, *cil* and *cia2*, respectively.


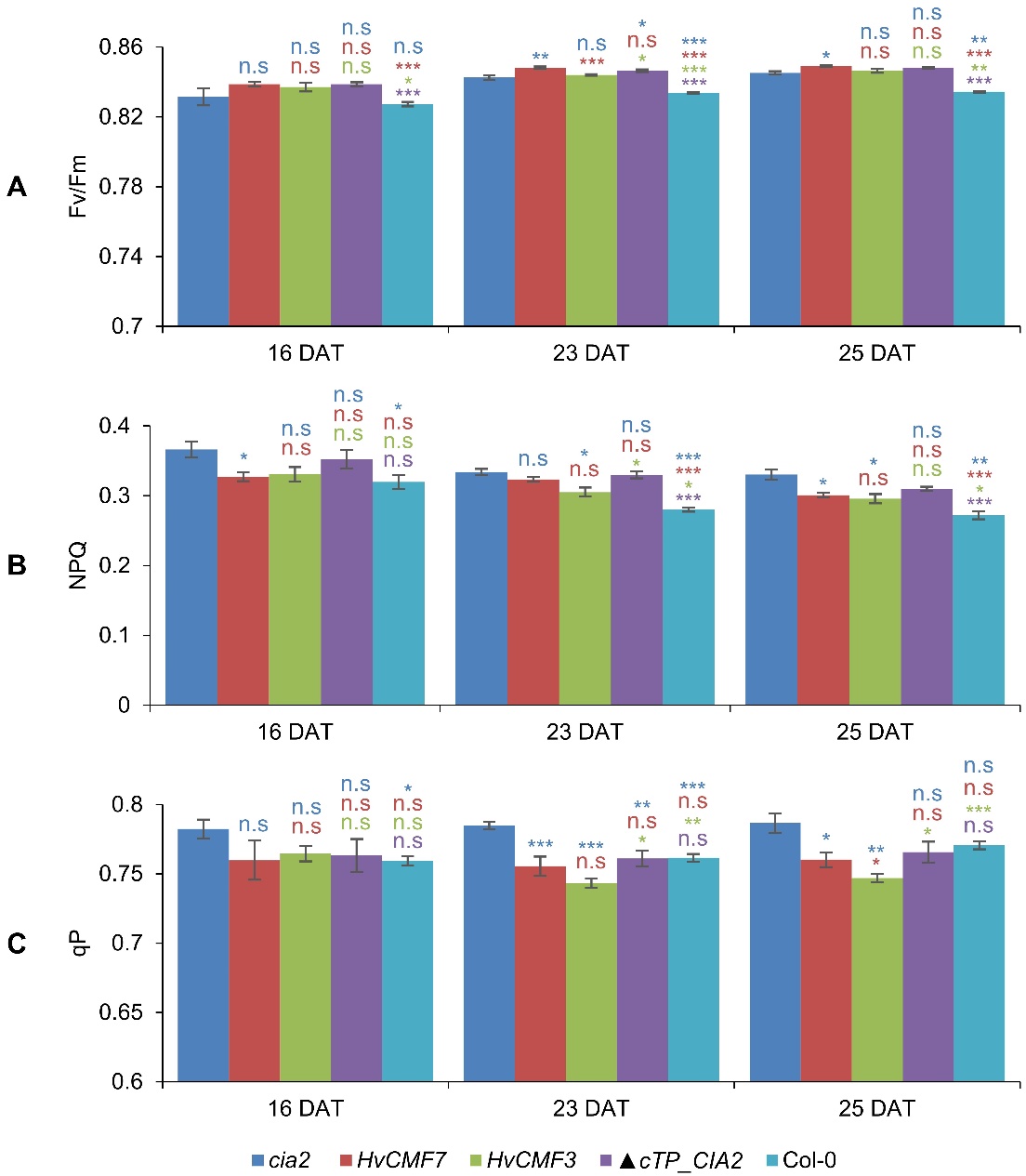


**Supplemental Figure 6. Measurement of photosynthetic performance of *cia2* mutant, complementation lines and Col-0.**

(A) Quantum yield of PSII photochemistry measured in the dark-adapted state.

(B) Non-photochemical quenching.

(C) Fraction of open PSII reaction centers based on the puddle model. Results are presented as mean ± SEM (N ≥ 9). *Student’s t-test* (Tails = 2; Type = 3) significant levels, n.s, not significant, * *p <* 0.05, * * *p <* 0.01, * * * *p <* 0.001. From top to bottom: statistical analysis of significance was performed as compared to *cia2*, *HvCMF7*, *HvCMF3* and *▲cTP_CIA2*, respectively. DAT, days after transfer to soil.


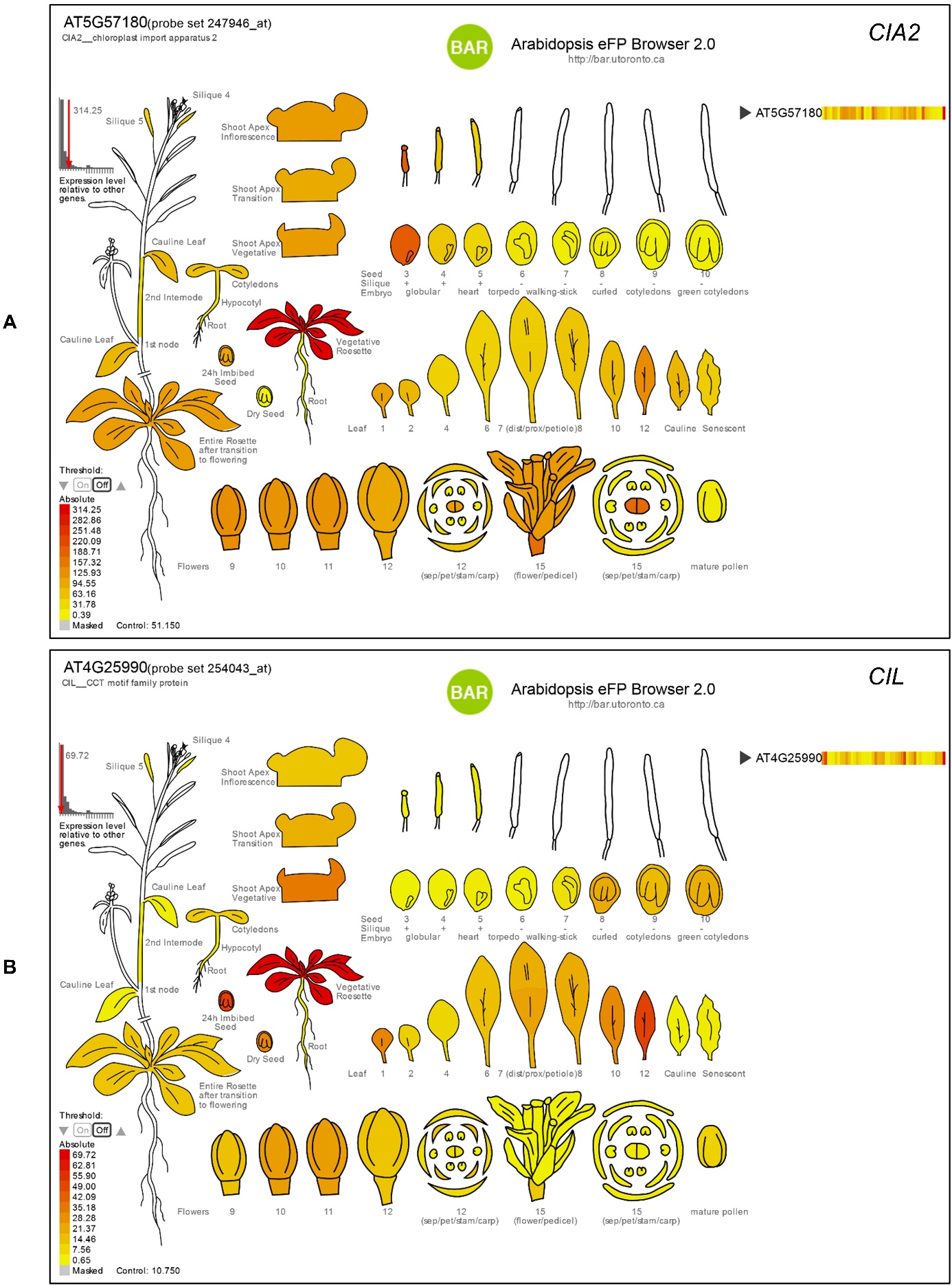


**Supplemental Figure 7. Expression profile of *CIA2* and *CIL* on eFP viewer (Winter et al., 2007).**

(A) Expression profile of *CIA2*.

(B) Expression profile of *CIL*.

| **Supplemental Table 1. Flowering time of Col-0, *cil*, *cia2* and *cia2cil*** | | | | | | | | | |
| --- | --- | --- | --- | --- | --- | --- | --- | --- | --- |
| **Genotype** | **Plant ID** | **7.11** | **8.11** | **9.11** | **10.11** | **11.11** | **12.11** | **13.11** | **14.11** |
| *cil* | 1942ML003 | - | Y | Y | Y | Y | Y | Y | Y |
| *cil* | 1942ML007 | Y | Y | Y | Y | Y | Y | Y | Y |
| *cil* | 1942ML011 | Y | Y | Y | Y | Y | Y | Y | Y |
| *cil* | 1942ML015 | - | Y | Y | Y | Y | Y | Y | Y |
| *cil* | 1942ML019 | Y | Y | Y | Y | Y | Y | Y | Y |
| *cil* | 1942ML023 | - | Y | Y | Y | Y | Y | Y | Y |
| *cil* | 1942ML027 | - | Y | Y | Y | Y | Y | Y | Y |
| *cil* | 1942ML031 | - | Y | Y | Y | Y | Y | Y | Y |
| *cil* | 1942ML035 | Y | Y | Y | Y | Y | Y | Y | Y |
| *cil* | 1942ML039 | Y | Y | Y | Y | Y | Y | Y | Y |
| *cil* | 1942ML043 | Y | Y | Y | Y | Y | Y | Y | Y |
| *cil* | 1942ML047 | Y | Y | Y | Y | Y | Y | Y | Y |
| *cil* | 1942ML051 | Y | Y | Y | Y | Y | Y | Y | Y |
| *cil* | 1942ML055 | Y | Y | Y | Y | Y | Y | Y | Y |
| *cil* | 1942ML059 | Y | Y | Y | Y | Y | Y | Y | Y |
| Col-0 | 1942ML001 | - | Y | Y | Y | Y | Y | Y | Y |
| Col-0 | 1942ML005 | - | Y | Y | Y | Y | Y | Y | Y |
| Col-0 | 1942ML009 | - | Y | Y | Y | Y | Y | Y | Y |
| Col-0 | 1942ML013 | Y | Y | Y | Y | Y | Y | Y | Y |
| Col-0 | 1942ML017 | - | Y | Y | Y | Y | Y | Y | Y |
| Col-0 | 1942ML021 | Y | Y | Y | Y | Y | Y | Y | Y |
| Col-0 | 1942ML025 | Y | Y | Y | Y | Y | Y | Y | Y |
| Col-0 | 1942ML029 | - | Y | Y | Y | Y | Y | Y | Y |
| Col-0 | 1942ML033 | - | Y | Y | Y | Y | Y | Y | Y |
| Col-0 | 1942ML037 | - | Y | Y | Y | Y | Y | Y | Y |
| Col-0 | 1942ML041 | Y | Y | Y | Y | Y | Y | Y | Y |
| Col-0 | 1942ML045 | - | - | - | Y | Y | Y | Y | Y |
| Col-0 | 1942ML049 | Y | Y | Y | Y | Y | Y | Y | Y |
| Col-0 | 1942ML053 | Y | Y | Y | Y | Y | Y | Y | Y |
| Col-0 | 1942ML057 | - | Y | Y | Y | Y | Y | Y | Y |
| *cia2* | 1942ML002 | - | - | Y | Y | Y | Y | Y | Y |
| *cia2* | 1942ML006 | - | - | - | - | - | Y | Y | Y |
| *cia2* | 1942ML010 | - | Y | Y | Y | Y | Y | Y | Y |
| *cia2* | 1942ML014 | - | Y | Y | Y | Y | Y | Y | Y |
| *cia2* | 1942ML018 | - | Y | Y | Y | Y | Y | Y | Y |
| *cia2* | 1942ML022 | - | - | - | - | Y | Y | Y | Y |
| *cia2* | 1942ML026 | - | - | - | Y | Y | Y | Y | Y |
| *cia2* | 1942ML030 | - | - | - | Y | Y | Y | Y | Y |
| *cia2* | 1942ML034 | - | Y | Y | Y | Y | Y | Y | Y |
| *cia2* | 1942ML038 | - | - | Y | Y | Y | Y | Y | Y |
| *cia2* | 1942ML042 | - | - | - | Y | Y | Y | Y | Y |
| *cia2* | 1942ML046 | - | - | - | Y | Y | Y | Y | Y |
| *cia2* | 1942ML050 | - | Y | Y | Y | Y | Y | Y | Y |
| *cia2* | 1942ML054 | - | Y | Y | Y | Y | Y | Y | Y |
| *cia2* | 1942ML058 | - | - | - | Y | Y | Y | Y | Y |
| *cia2cil* | 1942ML004 | - | - | - | - | - | - | Y | Y |
| *cia2cil* | 1942ML008 | - | - | - | - | - | Y | Y | Y |
| *cia2cil* | 1942ML012 | - | - | - | - | - | Y | Y | Y |
| *cia2cil* | 1942ML016 | - | - | - | - | - | - | Y | Y |
| *cia2cil* | 1942ML020 | - | - | - | - | - | - | Y | Y |
| *cia2cil* | 1942ML024 | - | - | - | - | - | - | Y | Y |
| *cia2cil* | 1942ML028 | - | - | - | - | - | - | - | Y |
| *cia2cil* | 1942ML032 | - | - | - | - | - | - | Y | Y |
| *cia2cil* | 1942ML036 | - | - | - | - | - | - | - | Y |
| *cia2cil* | 1942ML040 | - | - | - | - | - | - | - | Y |
| *cia2cil* | 1942ML044 | - | - | - | - | - | - | Y | Y |
| *cia2cil* | 1942ML048 | - | - | - | - | - | - | - | - |
| *cia2cil* | 1942ML052 | - | - | - | - | Y | Y | Y | Y |
| *cia2cil* | 1942ML056 | - | - | - | - | Y | Y | Y | Y |
| *cia2cil* | 1942ML060 | - | - | - | - | - | Y | Y | Y |
| -: Not yet bolting | | | | | | | | | |
| Y: Start bolting | | | | | | | | | |
| Y: First open flower visible | | | | | | | | | |

| **Supplemental Table 2. Primers used in this study.** | |
| --- | --- |
| Primer ID | Sequence (5'-3') |
| 1. Primers used for gRNA design and pSI57 cloning vector construction | |
| PS1-1_F | AAACCCGTCAAACAACACTCCTTC |
| PS1-1_R | ATTGGAAGGAGTGTTGTTTGACGG |
| PS1-2_F | AAACCCGTCAAACAACACTCCTT |
| PS1-2_R | ATTGAAGGAGTGTTGTTTGACGG |
| PS2_F | ATTGGCTTCGTCGTAATCACTGAG |
| PS2_R | AAACCTCAGTGATTACGACGAAGC |
| PS3_F | ATTGAGTTTTAGACGATTTCGACG |
| PS3_R | CAAACGTCGAAATCGTCTAAAACT |
| PromCasF | TAGAGGCCCTTAAGGCCTTAAAAAATTACGGATATGAATATAGGCATATCCG |
| PromCasR | CGTTTTCCTCGTTATCCAAGAAATCCTTATCCTTAATGATCTTG |
| CasTerm F | CTTGGATAACGAGGAAAACGAGGATATCTTGGAG |
| CasTerm R | CCTGCAGCCCGGGGGATCCAACTAGTAAGCCTATACTGTACTTAACTTGATTGC |
| 2. Primers used for check presence/absence of T-DNA | |
| HYG_F | CTCGGAGGGCGAAGAATCTC |
| HYG_R | TCGTCCATCACAGTTTGCCA |
| Cas9_F | GGTAGGTTCCGAGAGATGCG |
| Cas9_R | CACCAAGGCTCCACTCTCAG |
| 3. Primers used for genotyping | |
| AtCIL_F2 | TCCCAATCCAATCACTGCCC |
| AtCIL_R2 | TCCACCAGTTCTCGTCGTTG |
| 4. Primers used for subcellular localization | |
| AtCIL_F | CAACTAGTATGTCCTCTTGTGCCTATAGCTTCG |
| AtCIL_SC_R | TTCCCGGGCTAATCGTAGACCACTTAAATTCC |
| AtCIA2_F | CAACTAGTATGTCGGCGTGTTTAAGCAGCGGAG |
| AtCIA2_SC_R | TCCCCGGGCTCTTTGTCCACTTGGAGTGCTCTCA |
| 5. Primers used for functional complementation of *cia2* mutant | |
| HvCMF7_F | TCACTAGTATGGCGTCGTCCTGCATCCCGACGG |
| HvCMF7_R | GCAAGCTTGTCCTCCCCTAGGCCTCTTTCTCGCCGG |
| HvCMF3_F | CAACTAGTATGACGTCGTCTTGCATACCG |
| HvCMF3_R | CAAAGCTTCTAGCTCTCTTCCTCCAGGGC |
| AtCIA2_▲cTP_F | GAACTAGTATGACACAACGCAAAAGACCAAACCAGA |
| AtCIA2_▲cTP_R | TACCCGGGTTATCTTTGTCCACTTGGAGTGCTC |
| 6. Primer used for generating probes for Northern blot | |
| rrn23.T7 | GTAATACGACTCACTATAGGGAAGACTCGCTTTCGCTACG |
| rrn23.rp | CCTAGATGGCGAGAGTCCAG |
| rrn16.T7 | GTAATACGACTCACTATAGGGACCTTCCTCCGGCTTATCAC |
| rrn16.rp | ATGGATACTAGGCGCTGTGC |
| Red color indicates introduced restriction sites. | |
| Green color indicates introduced start codon or nucleotide added to guarantee in-frame translation. | |
